# Application of mitochondrial pyruvate carrier blocker UK5099 creates metabolic reprogram and greater stem-like properties in LnCap prostate cancer cells *in vitro*

**DOI:** 10.18632/oncotarget.5386

**Published:** 2015-09-25

**Authors:** Yali Zhong, Xiaoran Li, Dandan Yu, Xiaoli Li, Yaqing Li, Yuan Long, Yuan Yuan, Zhenyu Ji, Mingzhi Zhang, Jian-Guo Wen, Jahn M. Nesland, Zhenhe Suo

**Affiliations:** ^1^ Department of Oncology, The First Affiliated Hospital of Zhengzhou University, Zhengzhou, Henan Province, China; ^2^ Department of Gastroenterology, The Second Affiliated Hospital of Zhengzhou University, Zhengzhou, Henan Province, China; ^3^ Department of Pathology, The Norwegian Radium Hospital, Oslo University Hospital, University of Oslo, Montebello, Oslo, Norway; ^4^ Department of Pathology, Institute for Clinical Medicine, Faculty of Medicine, University of Oslo, Oslo, Norway; ^5^ Department of Surgery, The Affiliated Cancer Hospital of Zhengzhou University, Zhengzhou, Henan Province, China; ^6^ Department of Pathology, Capital Medical University, Beijing, China; ^7^ Department of Oncology, Henan Academy of Medical and Pharmaceutical Sciences, Zhengzhou University, Zhengzhou, Henan Province, China; ^8^ Department of Urology Surgery, The First Affiliated Hospital of Zhengzhou University, Zhengzhou University, Henan, China

**Keywords:** MPC blocker, mitochondrial dysfunction, glycolysis, stemness

## Abstract

Aerobic glycolysis is one of the important hallmarks of cancer cells and eukaryotic cells. In this study, we have investigated the relationship between blocking mitochondrial pyruvate carrier (MPC) with UK5099 and the metabolic alteration as well as stemness phenotype of prostatic cancer cells. It was found that blocking pyruvate transportation into mitochondrial attenuated mitochondrial oxidative phosphorylation (OXPHOS) and increased glycolysis. The UK5099 treated cells showed significantly higher proportion of side population (SP) fraction and expressed higher levels of stemness markers Oct3/4 and Nanog. Chemosensitivity examinations revealed that the UK5099 treated cells became more resistant to chemotherapy compared to the non-treated cells. These results demonstrate probably an intimate connection between metabolic reprogram and stem-like phenotype of LnCap cells *in vitro*. We propose that MPC blocker (UK5099) application may be an ideal model for Warburg effect studies, since it attenuates mitochondrial OXPHOS and increases aerobic glycolysis, a phenomenon typically reflected in the Warburg effect. We conclude that impaired mitochondrial OXPHOS and upregulated glycolysis are related with stem-like phenotype shift in prostatic cancer cells.

## INTRODUCTION

In the recent years, tumor cells exhibiting a metabolic reprogramming, known as the Warburg effect, has been attracting general attention in cancer research, wherein glycolysis is drastically upregulated with concomitantly increased lactate acid production even in the presence of oxygen [1]. Research done in the past decades has confirmed what Warburg observed and measured more than 90 years ago, was largely correct and nearly universally prevalent in cancer cells [2, 3]. Warburg concluded that upregulated glycolysis was likely due to dysfunctional mitochondrial oxidative phosphorylation (OXPHOS) [4]. Although this hypothesis has been challenged due to the findings that upregulated glycolysis in some cancers is not accompanied by detectable mitochondrial defects [5]. There actually exists a fine interplay between glycolysis and mitochondrial metabolism [6–8].

Recent studies have shown mutations in enzymes of the Krebs cycle in the etiology of some cancers, which suggest that dysfunctional mitochondria OXPHOS is a required step for cancer development at least in some cancer types [9, 10]. Multiple mechanisms attribute to dysfunctional OXPHOS, but it is already noticed that metabolism of pyruvate plays a central role [11].

Pyruvate, a critical molecule for mitochondrial OXPHOS, links the glycolytic pathway with the mitochondrial tricarboxylic acid cycle and plays an important role in this metabolic reprogramming. In most differentiated mammalian cells, pyruvate was processed from glucose in the cytosol and thereafter was directed into mitochondrial and oxidized for efficient ATP production. While in cancer cells, pyruvate was converted to lactate by aerobic glycolysis in most cases, the so-called Warburg effect, which is inefficient but seems to enable cancer cell proliferation [12].

Pyruvate enters mitochondria through a recently identified mitochondrial pyruvate carrier (MPC), a 150-kDa complex of MPC1 and MPC2 located on the inner mitochondrial membrane, which transports pyruvate across the inner mitochondrial membrane and to the mitochondrial matrix [13–15]. Absence of either MPC1 or MPC2 leads to a loss of mitochondrial pyruvate uptake and utilization [16]. It is well known that UK5099 (acyano—(1-phenylindol-3-yl)-acrylate is an inhibitor of MPC, which inhibits the MPC by specifically modifying a thiol group on the carrier [17].

Two studies demonstrated that the undifferentiated stem cells showed suppressed mitochondrial activity [18, 19]. Vazquez-Martin A reported induced pluripotent stem cells (iPS) exhibit a glycolytic metabolic phenotype [20]. Given the observation that decrease in pyruvate oxidation may be associated with the Warburg effect, and different types of stem cells exhibit a glycolytic metabolic phenotype [21, 22], we asked whether reduced MPC activity with UK5099 resulted in aerobic glycolysis and was correlated with high stemness phenotype of prostatic cancer cells. We are probably the first to use UK5099 to block MPC in prostate cancer cells, in consideration of its effect on cell stemness. We assessed its effect on metabolic phenotypes and stemness-like features. It was found that UK5099 enhanced aerobic glycolysis and decreased mitochondrial OXPHOS in LnCap cell *in vitro*. This was accompanied by increasing proportion of side population (SP) fraction, higher level of stemness markers expression and exhibiting high resistance to chemotherapy. Our results verify that MPC blocker UK5099 enhances stem-like phenotype of prostatic cancer cell through metabolic shift from OXPHOS to aerobic glycolysis.

## RESULTS

### UK5099 reduced mitochondrial pyruvate concentration

**Figure 1:**
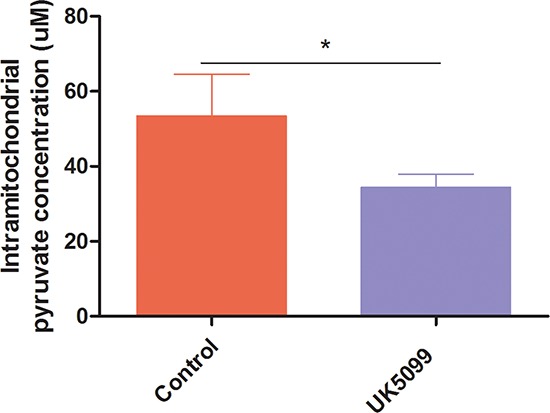
UK5099 blocked pyruvate transportation into mitochondrial in LnCap cells *in vitro* Mitochondrial pyruvate concentrations were tested by using pyruvate assay kit to determine whether pyruvate transportation into mitochondrial was blocked. Data were expressed as mean ± SD. *vs control *p* < 0.05.

As an initial test of whether UK5099 inhibited mitochondrial pyruvate transportation in prostate cancer cell line LnCap, mitochondrial pyruvate concentration of LnCap cells treated with UK5099 was determined firstly. As shown in Figure 1, application of UK5099 significantly reduced the pyruvate concentration in mitochondria (*p* = 0.03). It was therefore confirmed that UK5099 acts as a MPC inhibitor in LnCap cells.

### UK5099 suppressed proliferation and arrested cell cycle at G1/G0

**Figure 2:**
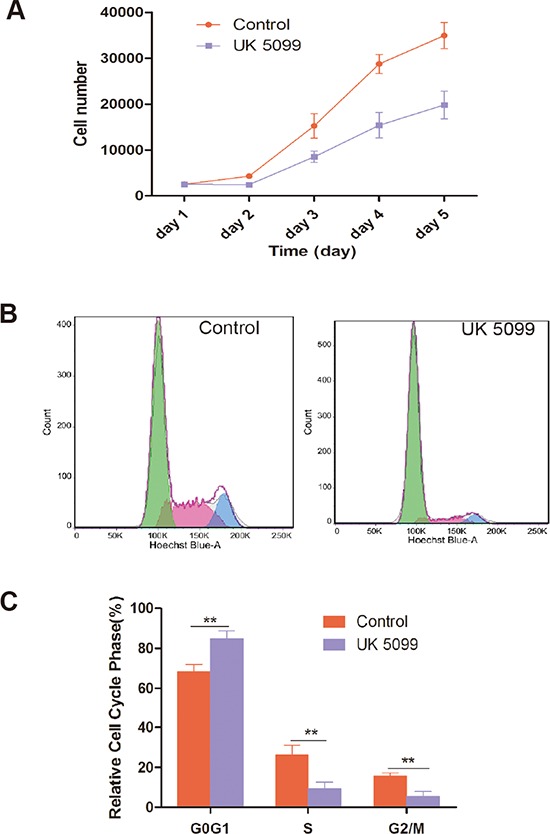
UK5099 inhibited cell proliferation and changed cell cycle **A.** UK5099 inhibited LnCap cell proliferation. The number of cells was counted every day from the 1st to 5th day. Data points are the means with standard deviations from three independent experiments. **B.** LnCap cells treated with UK5099 and control cell were subjected to cell cycle analysis by FACS with representative images of four separate experiments. **C.** Cell cycle distribution of UK5099 treated control LnCap cells. The cell cycle distribution was calculated and expressed as mean ± SD of three separate experiments. * vs control *p* < 0.05;** vs control *p* < 0.01.

Cell growth of prostate cancer cell line LnCap treated with UK5099 was evaluated by cell counting at variable time points. Growth curves were also generated. As shown in Figure 2A, UK5099 suppressed the cell proliferation in LnCap cells.

The effect of UK5099 on cell cycle distribution of LnCap cells was further analyzed. As shown in Figure 2B, and 2C, LnCap cells treated with UK5099 show significantly increased percentage of G1/G0 phase (*P* = 0.0067) and decreased percentage of S (*P* = 0.0084) and G2/M phases (*P* = 0.0031) compared with the control cells, indicating an effect of G0/G1 phase arrest of UK5099 on LnCap cells.

### UK5099 promoted mitochondrial dysfunction and enhanced aerobic glycolysis

**Figure 3:**
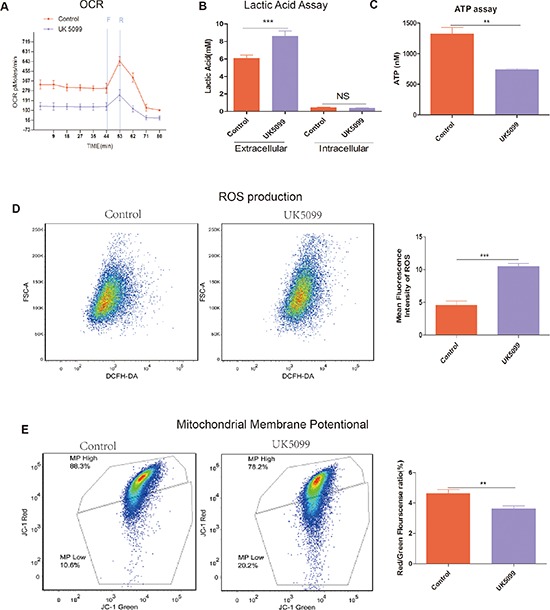
UK5099 attenuated mitochondrial function and increased glycolysis **A.** OCR (pmol/min/50000 cells) measurements were obtained at baseline and by adding carbonylcyanide-p-trifluoromethoxyphenylhydrazone (FCCP, F, 400 nM) to uncouple the mitochondria for maximal OCR and Reteno(R, 1 mM). **B.** UK5099 increased extracellular lactate acid level and had no effect on intracellular lactate acid. Lactate level was determined by lactate assay kit. Total ATP levels were expressed as mmol/10^6^cells. Data were shown as mean ±SD. **C.** UK5099 decreased ATP production. ATP production was obtained by using ATP assay kit. Data were shown as mean ± SD (nmol/10^6^). **D.** Significantly higher level of ROS in UK5099 treated cells. Left panel shows representative ROS flow cytometry graphs while the right panel shows histograms of the mean fluorescence intensities of ROS obtained with microplate reader. Data were expressed by mean fluorescence ± SD. **E.** UK5099 decreased mitochondrial membrane potential (ΔΨm) in LnCap cell. ΔΨm was measured with a unique cationic dye of 5,5′,6,6′-tetrachloro 1,1′,3,3′-tetraethylbenzimidazolcarbocyaenina iodide (JC-1) and analyzed with a flow cytometer as shown in Materials and Methods. * vs control *p* < 0.05; ** vs control *p* < 0.01; ***p < 0.001.

To gain insight into the mitochondrial OXPHOS and relative utilization of glycolysis in UK5099 treated cells, oxygen consumption rate (OCR), ATP, reactive oxygen species (ROS) production and mitochondrial membrane potential (ΔΨm) were examined (Figure 3).

### OCR

OCR in UK5099 treated cells was examined using a Seahorse XF-24 extracellular flux analyzer. OCR under basal conditions and maximal respiration in the presence of FCCP (an uncoupling agent that allows maximal electron transport) are shown in Figure 3A. Basal cellular OCR and maximal OCR of UK5099 treated cells were found to be significantly lower than that in the control cells Figure 3A. In the presence of FCCP, a concomitant increase of 244.6 ± 27.4 pmol/min in OCR was observed in the control cells, while there was only a slight increase (103.9 ± 48.6 pmol/min) in the UK5099 treated cells. The increase was considered as respiratory reserve capability, i.e. maximal OCR minus basal OCR. This implies that at basal levels, the UK5099 treated cells were operating closer to maximal OCR capacity, which resulted in a lower reserve capacity. Lower respiratory reserve capacity is also linked to lower mitochondrial fidelity. We were able to identify that UK5099 promoted defective mitochondrial function, indicated by low basal OCR and a lack of response to FCCP.

### ATP production and lactic acid level

To understand whether there was ATP generation alteration in the cells treated with UK5099, we next analyzed ATP production. As shown in Figure 3C, cells treated with UK5099 produced significantly less ATP (*p* = 0.0013). Next we sought to characterize the glycolytic rate in these cells. We used EnzyChrome lactate assay kit to determine the medium and cellular lysate lactic acid levels. It was found that extracellular lactic acid production increased significantly in the medium while intracellular lactic acid production was not influenced (Figure 3B), indicating a higher glycolytic efflux in the cells treated with UK5099 which tend to rely on glycolysis, thus produced more lactic acid.

### ROS generation

To provide evidence about mitochondria function influence when treated with UK5099, we sought to determine whether UK5099 could directly affect ROS production in LnCap cells. As shown in Figure 3D, ROS production in LnCap cells treated with UK5099 is about 2 times higher than that in the LnCap control cells (*p* < 0.001), further indicating that LnCap cells treated with UK5099 show dysfunctional mitochondria in contrast to the LnCap control cells.

### Mitochondrial membrane potential (ΔΨm)

ΔΨm is an important measure of how the mitochondria are functioning [24]. ΔΨ of LnCap cells treated with UK5099 was assessed using JC-1. As shown in Figure 3E, the ΔΨm of LnCap cells treated with UK5099 is significantly lower than that in the LnCap control cells.

All the above results showing lower OCR, ATP production, ΔΨm and higher of extra cellular lactic acid efflux and higher ROS production indicate that UK5099 treatment result in mitochondrial OXPHOS dysfunction and the UK5099 treated LnCap cells are forced to rely on glycolysis to survive, even in the presence of normal oxygen concentration (normoxia).

### UK5099 increased side population (SP) and the expression of stemness markers

**Figure 4:**
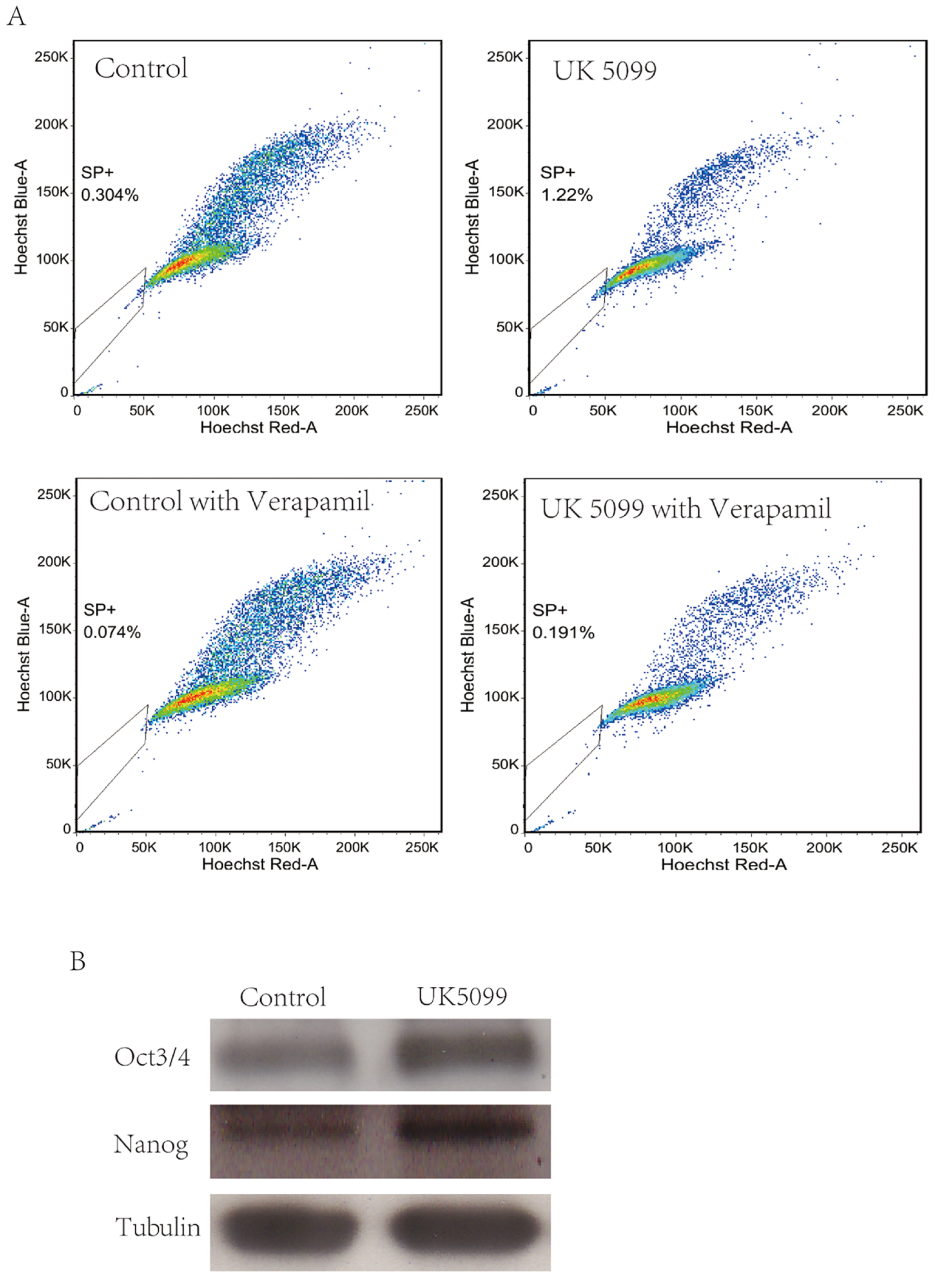
UK5099 increased side population cells and stemness markers in LnCap cell **A.** UK5099 treatment increases the side population in LnCap cells. Side Population was analyzed through uptake of Hoechst33342 with or without the presence of verapamil. **B.** Western blot analysis of stem cell markers (Oct3/4 and Nanog) in control cells and UK5099 treated cells.

We tested whether the MPC blocker could increase the proportion of the SP in LnCap cells by using a hoechst-extrusion assay. The results revealed that the proportion of the SP was significantly increased in the UK5099 treated cells compared to the control cells Figure 4A, (*p* = 0.005). In the presence of verapamil, the increase in SP was largely abrogated, suggesting that ABCG2 is at least partially responsible for the increase in side population, although it remains possible that other drug pumps could play a role in UK5099-treatment induced side population. Compared to the controls, protein expression of Oct3/4 and Nanog was upregulated in the UK5099 treated LnCap cells as well Figure 4B.

### UK5099 decreased chemosensitivity of LnCap cell

**Figure 5:**
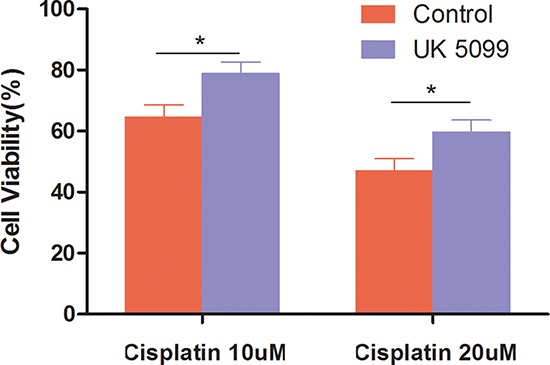
Cells treated with UK5099 tended to be highly resistant to cisplatin Cells were incubated for 48 hours in the presence of increasing cisplatin concentrations. Cell numbers were counted and cell viability was determined based on non-treated cell. *vs control *p* < 0.05.

To evaluate whether UK5099 treatment could affect the sensitivity of chemotherapy, cell viability examination was conducted in the UK5099 treatment and control cells after 10 μM or 20 μM of cisplatin was added for 72 hrs. As shown in Figure 5, all cells showed reduced cell viability when cisplatin concentration was increased from 10 μM to 20 μM. However, UK5099 treated cells always displayed significantly increased cell viability than the control cells (*p* = 0.0105 and 0.0175 for 10 μM and 20 μM cisplatin treated cells respectively), highlighting significant chemotherapy-resistance of the LnCap cells treated with UK5099.

## DISCUSSION

*MPC1* and *MPC2* gene expression has been investigated in cell lines like embryonal, heatologic, ovarian and colorectal cancer cell lines. The expression levels are variable depend on different cell lines [25]. *MPC1* and *MPC2* gene expressions were assessed in prostatic cancer cell lines LnCap, DU145 and PC3 firstly in our lab, and it was found that LnCap exhibited higher expression levels of MPC1 and MPC2 compared to DU145 and PC3 (Data not shown). We therefore applied LnCap cell line for further study in this project. The comparatively higher level of MPC1 and MPC2 expressions in LnCap cells may indicate rather better mitochondrial function in these cells compared to the DU145 and PC3 cells, since both DU145 and PC3 cells are more malignant than LnCap cells.

In our study, it was confirmed that UK5099 blocked pyruvate transportation into mitochondria in LnCap cells by analyzing the mitochondrial pyruvate concentrations. Upon UK5099 application, OCR, ATP production and membrane potential were significantly decreased and the extracellular lactate acid efflux and ROS production were significantly increased in the UK5099 treated cells. All results are in line with impaired mitochondrial OXPHOS status and enhanced aerobic glycolysis in the UK5099 treated LnCap prostatic cancer cells.

The Warburg effect or named as aerobic glycolysis wherein glycolysis was upregulated even in the presence of oxygen is now recognized as an important hallmark of cancers. Research done in the past 15 years has confirmed the prevalence of Warburg effect [5, 11, 26]. Warburg also hypothesized that the increased glycolysis was due to mitochondrial dysfunction [4]. However, the mechanisms leading to the increased rate of glycolysis are debatable and only partially understood currently. But there actually exists a fine interplay between glycolysis and mitochondrial metabolism as reported in literature. It was discovered that suppressing aerobic lactate production by stably knocking-down lactate dehydrogenase directly impacted mitochondrial respiration. Cells with decreased LDH-A activity showed an increase in oxygen consumption and OXPHOS activity [6, 7]. It was also clear that acute overexpression of lactate dehydrogenase-A could perturb cell mitochondrial metabolism and insulin secretion [6, 7].

Warburg-like metabolic changes were once considered as a mere consequence of the aberrant cancer cell growth, while in the recent research, it was demonstrated that this metabolic reprogramming has cancer-causing activity [27]. Yi Zhu and his group identified that the nucleophosmin (NPM1) can promote Warburg effect. NPM1 was also found up-regulated in pancreatic cancer, and indicated a poor prognosis in their study [28]. While in other hand, the results of a colon cancer model study reported by Bianchi et al imply that glucose and amino acid deficiency conditions imposed by short term starvation promote an anti-Warburg effect characterized by increased oxygen consumption but failure to generate ATP, resulting in oxidative damage and apoptosis [3]. RRAD, a small GTPase, was found to repress the Warburg effect through negatively regulating the NF-κB signaling and inhibit the GLUT1 translocation. The expression of RRAD is frequently down-regulated in lung cancer and is associated with tumor progression and poor prognosis as Liu J et al reported [29].

Mitochondria are a central hub for biosynthesis of amino acids, nucleic acids and lipids, and are the main providers of NADH and NADPH. They play a key role in the context of cancer metabolic reprogramming. Many vital cellular parameters are controlled by mitochondria. These include regulation of energy production, generation of ROS, and maintaining mitochondrial membrane potential and so on. Defects in mitochondria, caused by mtDNA depletion or others, can result in metabolic reprogram, which is in accordance with our result. For example, Yu M et al showed that breast tumor cell line lacking mtDNA generated increased levels of lactate with concomitantly reduced oxygen consumption and ATP production[30].

Defects in mitochondrial metabolism can initiate a complex cellular reprogramming that supports tumor initiation and growth [31, 32]. Increasing evidence has shown that mitochondrial dysfunction provides survival advantage to cancer cells [31, 33–35]. In addition, mtDNA-depleted cells display a decreased sensitivity to chemotherapeutic drugs and resistant to apoptosis induced by staurosporine and anti-Fas antibody [30].

There are indications that mitochondrial function is associated with cell stemness. Changes in the parameters like ROS and mitochondrial membrane potential, which controlled by mitochondrial function, can impinge on biosynthetic pathways, cellular signal transduction pathways, transcription factors and chromatin structure to shift the cells between a quiescent, differentiated state and an actively proliferating one [24]. As a critical molecule for mitochondrial OXPHOS, modulation of pyruvate metabolism is vital for the maintenance of stem cell populations [36, 37]. It is reported that antimycin A was used to inhibit complex III of the mitochondrial respiratory chain, indicating that the inhibition of mitochondrial OXPHOS may prevent differentiation of human embryonic stem cell [3]. Mitochondrial function of iPS cells is drastically altered during the process in acquiring embryonic features, which impacts the cellular bioenergetics profile wherein the metabolism is shifted from OXPHOS to glycolysis and returns to OXPHOS during subsequent iPS cells differentiation [20].

We have observed that UK5099 has the ability to expand the side population. In theory, the cells within a population that display higher efflux of DNA-binding dye Hoechst 33342 via ABCG2 constitute the SP. In multiple cancers these cells have been observed to be more cancer stem cell-like. Thus, UK5099-induced expansion of the SP could serve as an evidence of a potentially novel paradigm in which UK5099 promotes a cancer stem cell phenotype. We also performed Western blotting of stemness markers Nanog and Oct3/4 in this study. It was found that cells treated with UK5099 tended to express higher level of Nanog and Oct3/4. We next analyzed the chemo-sensitivity to cisplatin and found that cells treated with UK5099 were more resistant to cisplatin. These data strongly suggest that the metabolic shift caused by UK5099 is related to cell stemness upregulation.

In keeping with our results, MPC1 and MPC2 are reported expressed at a very low level in embryonic stem cells, and their expression increases upon differentiation [46, 47]. MPCs (particularly MPC1) were underexpressed in many cancers and low expression correlates with poor survival as reported by John C. Schell [25]. When using the opposite approach, re-expressed MPC in colon cancer cells, it was confirmed with an anti-Warburg effect in which OXPHOS was enhanced and glycolysis was decreased. Accompany with this, less stem cell features were also observed, like defects in soft agar, spheroid, xenograft growth and lose of stemness markers[25].

If pyruvate transportation into mitochondria is blocked by UK5099, pyruvate will be forced to convert to lactate in the cytoplasm. The cellular lactate will be effluxed out of the cells under conventional condition. It is known that MPC inhibitor UK5099 may also inhibit proton-linked plasma membrane monocarboxylate transporters (MCTs) that carry lactate and pyruvate across biological membranes. However, we discovered in our study that after UK5099 application, the extracellular lactate production was significantly increased while the intracellular lactate production was not influenced. Theoretically, the inhibition of MTCs by UK5099 may influence the efflux of lactate, so that extracellular lactate after application of UK5099 may not increase. This may be explained by the finding that UK5099 is approximately 300-fold more potent at inhibiting the MPC compared to the monocarboxylate transporters as reviewed by Gray et al in 2014 [48], so that the lactate efflux influence of UK5099 used in our study is negligible, at least in these LnCap cells.

In conclusion, we show here that impaired import of pyruvate via blocking MPC by UK5099 resulted in mitochondrial OXPHOS dysfunction and enhanced aerobic glycolysis concomitant with upregulated cell stemness features such as increased proportion of SP cells, higher levels of stem cell markers and highly resistant to chemotherapeutic reagent cisplatin. We propose that MPC blocker (UK5099) application may be an ideal model for Warburg effect studies in prostatic cancer cells, since it attenuates mitochondrial OXPHOS and increases aerobic glycolysis, and most likely this metabolic shift confers enhanced stemness phenotype and increased survivability of prostatic cancer cells. Therefore, targeting this axis may offer a novel opportunity for selective anti-cancer therapy.

## MATERIALS AND METHODS

### Cells and culture conditions

Prostatic cancer cell line LnCap was obtained from ATCC (American Type culture collection, USA) and maintained in our laboratory for the study. The cells were cultured in RPMI 1640 medium (Gibco-BRL) supplemented with 10% fetal bovine serum (FBS, Gibco), 100 U/ml of penicillin and 100 ug/ml streptomycin at 37°C, and 5% CO2. UK5099 (PZ0160, Sigma, St. Louis. MO, USA), a chemical that was known as MPC blocker, was added to a final concentration of 10 μM to reduce pyruvate transportation into mitochondrial. This concentration of 10 μM was achieved after performing proliferation assay, pyruvate assay and lactic acid assay with different concentrations of UK5099 ranging from 10 μM to 100 μM. It was noticed that LnCap cells grew more slowly when exposed to higher concentrations (50, 100 μM) UK5099, while the pyruvate mitochondrial concentrations and lactic acid levels remained at almost the same level. Therefore, 10 μM UK5099 was regarded as an appropriate concentration for subsequent experiments in this study.

### Measurement of mitochondrial pyruvate concentration

Cells were seeded evenly into three 10 cm culture plates at a density of 5 × 10^6^ cells/plate and cultured for 12 hours. Cells were harvested when grew at 75% confluent, and mitochondria were isolated by performing the first part of mitochondrial DNA isolation kit steps (Biovision K280-50, SFO, USA). 50 μL lysis buffer was added to the isolated mitochondria and homogenized by sonication. Pyruvate concentration was determined by pyruvate assay kit (BioAssay Enzychrom EPYR-100, CA, USA) according to the instructions. Briefly, 90μl working reagent (including enzyme mix and dye reagent) was added to each 10μl of sample or standard before. Tap plate to mix and incubated 30 min at room temperature. The color intensity of the reaction product at 570 nm is directly proportional to pyruvate concentration. Pyruvate concentrations of samples were calculated based on OD values of standards.

### Lactate assay

1*10^6^ cells were seeded into 6 cm culture dishes and cultured for 24 hours. The culture medium was centrifuged and ready to go lactate assay. Those cells were harvested in 200 μl PBS to achieve cell lysate by sonication (in ice-water bath) before being centrifuged at 12,000 g for 10 min. The clear supernatant was applied for lactate assay.

The lactate assay was performed using an EnzyChrome lactate assay kit (BioAssay, ECLC-100, CA, USA) according to the manufacturer's instructions. 80 ul working reagents (including lactate dehydrogenase which catalyzed oxidation of lactate, NADH formed in this reaction reduces a formazan reagent) were added to each standards or samples. The intensity of the product color, measured at 565 nm, is proportionate to the lactate concentration. The sample L-lactate concentrations were determined based on OD values of samples and standards. Experiments were conducted in triplicate.

### Cell growth assay and cell count

Cells were seeded at a density of 2500 cells/well in 6-well plates (NUNC™, Thermo Scientific, Denmark). The cells in different wells were harvested every 24 hours using 0.25% trypsin (Invitrogen) and counted by Countess® Automated Cell Counter (Life Technologies). 10 μl of single cell suspension was mixed well with 10 μl of 0.4% trypan blue dye. 10 μl of the mixture was loaded onto a cell counting chamber (Life Technologies) and the chamber was applied into Countess® Automated Cell Counter for cell counting.

### Cell cycle analysis

For analysis of cell cycle phase distribution, cells were fixed in 70% ethanol, treated with RNase A and stained with DAPI. DNA contents (50,000 cells per sample) were measured with an LSRII flow cytometer (Becton Dickinson, San Jose, CA, USA).

### OCR measurement using seahorse xf24-extracellular flux analyzer

Oxygen consumption rate (OCR) was measured using a Seahorse XF24 extracellular flux analyzer (Seahorse Bioscience, North Billerica, MA, USA). Briefly, cells were plated at 5*10^4^Cells/well in a XF24 cell culture microplate (Seahorse Bioscience) and cultured for 6 hours for cells to attach to the bottom. The cells were switched into unbuffered DMEM supplemented with 2 mM sodium pyruvate and 20 mM carnosine 1 h prior to the beginning of the assay and maintained at 37°C. OCR was reported in the unit of picomoles per minute. After baseline measurements, OCR was measured by sequentially adding to each well carbonylcyanide-p-trifluoromethoxyphenylhydrazone (FCCP) and rotenone, to reach working concentrations of 0.4 μM and 1 μM, respectively.

### Determination of atp production

1*10^6^ cultured cells were harvested in 200 μl PBS and cell lysate was achieved by sonication (in ice-water bath) before the homogenate was centrifugated at 12,000 g for 10 min. Finally, in 96-well plates, 10 μl of the supernatant was mixed well with 100 μl of luciferase reagent, which catalyzed the light production from ATP and luciferin ATPliteTM Luminescence Assay kit (PerkinElmer) according the manufacturer's instructions. Luminescence was measured by luminometer (Fluroskan Ascent FL, Thermo). Standard curve was also generated and the amount of ATP in the experiments samples was calculated. Total ATP levels were expressed as nmol/10^6^ cell.

### Determination of ros production

Total ROS production was investigated using dichlordehydrofluorescein-diacetate (DCFH-DA; Molecular Probes). Briefly, cells in approximate 60% confluence were washed with PBS and stained with 10 μM DCFH-DA in FluoroBrite™ DMEM medium (contains 10% FBS and pre-warmed to 37°C) for 20 min in CO2 incubator at 37°C before dissociated with TrypLE™ Express. The cells (30, 000 cells per sample) were analyzed with a flowcytometer FACScan (Becton Dickinson, San Jose, CA). The signal was excited with 488 nm laser and detected with band pass filter set 530/30. Unstained samples of each group were prepared to measure background fluorescent signal. Cells treated with 250 μM hydrogen peroxide were performed as positive controls.

The ROS production was also verified using a fluorescence microplate reader. The cells were incubated with 10 μM DCFH-DA at 37°Cfor 20 min. After the incubation the cells were washed and re-suspended in PBS. 1*106 cells per sample were added in 96-well microplate (E&K Scientific, EK-25075) and the fluorescence was measured with an excitation wavelength of 485 nm and emission wavelength of 532 nm using a fluorescence microplate reader (μQuant; Bio-Tek Instruments, Winooski, VT, USA).

### Measurement of mitochondrial membrane potential (ΔΨm)

ΔΨm was measured with a unique cationic dye of 5,5′,6,6′-tetrachloro 1,1′,3,3′-tetraethylbenzimidazolcarbocyaenina iodide (JC-1). The normally green fluorescent dye forms red fluorescent aggregates when concentrated in energized mitochondria in response to their higher membrane potential. Briefly, cells were stained with JC-1 (1 μM) at 37°C for 30 min. After incubation the cells were washed twice and resuspended in PBS before analyzed (30,000 cells per sample) with a flow cytometer (FACScan, Becton Dickinson, San Jose, CA). For each cell the average intensity of green and red fluorescence was determined, and the ratio of JC-1 aggregate (red) to monomer (green) was calculated. A decrease in this ratio was thus interpreted as a decrease in mitochondrial membrane potential[23].

### Flow cytometry analysis for Hoechst efflux (side population assay)

1*10^6^ cells per sample were prepared and stained with 5 μg/mL of Hoechst 33342 (stock solution of 1 mg/ml; Sigma) in 2 mL PBS containing 2% bovine serum albumin (BSA) at 37°Cfor 120 min. After incubation, the cells were washed and resuspended in PBS. In addition, 2 μg/mL of propidium iodide (PI) was added for dead cell discrimination. For each sample, 30,000 cells were analyzed for hoechst efflux examination using the same FACScan flowcytometer as described above., Verapamil (50 μM; Sigma) was used to block Hoechst efflux and determine the area of the side population in a separate sample.

### Chemosensitivity assay

Cells were seeded in 25 cm^2^ flasks at a density of 1*10^6^ cells per flask and allowed to attach for 12 h. Various gradient concentrations of cisplatin (0, 10, 20 μmol/L) were added to each flask. After 72 h, Cells were brought into suspension using trypsin and counted in triplicate samples. Cell viability was calculated based on the survival of non-treated cells. The experiments were conducted in triplicate.

### Western blotting assay

Briefly, 50 μg of cellular proteins were loaded onto a 10% SDS-PAGE. After electrophoresis, the proteins were transferred to PVDF membranes (BIO-RAD, USA). Membranes were blocked in 5% milk, washed with PBST (PBS with 0.1% Tween), and incubated with the indicated antibodies. After washing and incubation with horseradish peroxidase-conjugated anti-rabbit or anti-mouse antibody at 1:1000 in 5% milk, the membranes were washed, and bound horseradish peroxidase was detected by enhanced chemiluminescence (GE Healthcare, UK) and exposed to X-ray film. The optimized antibodies used in this study included: Oct3/4 (1 μg/ml, MAB1759, R&D), Nanog (1 μg/ml, AF1997, R&D), Tubulin (1:5000, 103M4773, Sigma).

### Statistical analysis

All data represented at least three independent experiments and statistical analyses were performed using Graphpad Prism 5. Data were analyzed by the one-way ANOVA test and student's t test (*p* < 0.05 was considered statistical significant).
